# Effectiveness of cognitive-behavioural group therapy for inpatients

**DOI:** 10.1186/1745-0179-2-16

**Published:** 2006-07-21

**Authors:** Franco Veltro, Ian Falloon, Nicola Vendittelli, Ines Oricchio, Antonella Scinto, Antonella Gigantesco, Pierluigi Morosini

**Affiliations:** 1 Dipartimento di Salute Mentale, ASL 3 "Centro Molise", Campobasso, Italy; 2 Department of Psychiatry University of Auckland, New Zealand; 3 Reparto Salute Mentale, Centro Nazionale di Epidemiologia, Sorveglianza e Promozione della Salute, Istituto Superiore di Sanità, Roma, Italy

## Abstract

**Objective:**

To measure the effectiveness of manualized cognitive-behavioural group therapy (CBGT) when it is integrated into the routine care on a general hospital psychiatric inpatient unit.

**Methods:**

A pre-post design is used to measure the "process", "results" and "outcome" indicators in the year before CBGT was introduced (2001) in contrast to the subsequent two years (2002, 2003). Readmission to hospital, compulsory admissions, ward atmosphere (i.e. the use of physical restraint, episodes of violent behaviour) and patients' satisfaction were assessed.

**Results:**

90% of all inpatients in the years 2002–2003 attended the group therapy. In the years after CBGT was introduced the rate of readmission declined from 38% to 27% and 24% (p < .04), compulsory admissions were reduced from 17% to 4% (p < .03), the ward atmosphere and patients' satisfaction were both excellent (p < .01).

**Conclusion:**

It is probable that the improvements observed were attributable to the group therapy. These results and those observed in an earlier study are promising and further investigations of this approach are indicated.

## Background

Cognitive-behavioural therapies have been applied successfully for schizophrenia in outpatient settings for many years. Family and skills training strategies have shown benefits in reducing clinical and social morbidity [1,2], while recent research on individual cognitive-behavioural therapy has led to a classification of "probably effective" in reducing persisting psychotic symptoms [3-6]. Applications in acute inpatient settings have been rare and have involved highly selected cases [7-9].

Despite a strong "socio-therapeutic" orientation no psychosocial approach to inpatient treatment has been developed in Italy [10]. Inpatient treatment tends to be crisis-oriented, with the aim to control positive symptoms and to discharge the patients as soon as possible to community mental health centres. Frequently the approach followed in the ward (mainly pharmacological) is different from that of the community-based service (mainly psychosocial). This creates considerable confusion for patients.

Several inpatient units have introduced group-therapy interventions, most of them psychodynamic, but without evaluation of their efficacy. Two preliminary studies have evaluated the benefits of inpatient educational group therapy [11,12]. Bazzoni and his colleagues [10] have recently conducted **Cognitive Behavioural Group Therapy **(CBGT) that was based on the stress-vulnerability theory at an inpatient unit in Rome. The main characteristics of this approach are daily psychoeducation and problem solving aimed at identifying and reducing biomedical and psychosocial factors associated with hospital admission and improving adherence to continued treatment. On the basis of their initial positive results we began collaborating with this group to provide clearer structure to the methods and to write a manual [13].

In this paper we describe the approach and the results of a 2-year follow-up of the intervention program that has been a core part of our inpatient program since 2001.

## Methods

### Description of cognitive behavioural group therapy for inpatients

The aims of Cognitive Behavioural Group Therapy, CBGT, for inpatients are:

a) general: to improve the collaboration of patients with biopsychosocial treatment programs and to decrease the number of violent and aggressive behavioural acts;

b) for services: 1) to motivate and to improve the professional skills of nurses and to minimize *burn-out*; 2) to improve collaboration between patients and among patients and professionals; 3) to assist patients and staff to cope **with acute mental illness.**

c) for patients: 1) to give a dimensional view of illness (i.e., these are problems experienced by the majority of people) and to normalize their experience; 2) to reduce the isolation of inpatients by sharing their experience of their disorders; 3) to increase compliance in the ward and after discharge; 4) to improve the ability to recognize the early warning signs of exacerbations; 5) to improve self-control and self esteem.

### Characteristics of the intervention

The program is based on the vulnerability/stress model that postulates that major episodes of mental disorders occur when patients have increased biological vulnerability and/or high levels of environmental stress that exceed their coping capacities [14]. Sessions are provided to teach patients to recognize events and situations that they find very stressful and to recognize their early warning signs of stress overload and impending recurrences. The importance of optimal adherence to **medication**, especially at times of stress is emphasized, as well as seeking effective strategies to cope with specific **stressors**. Practical training is provided in effective interpersonal communication and structured problem solving skills. Patients are helped to clarify their personal goals and plan for adjustments after they leave hospital. These include any problems they might encounter accessing and accepting outpatient treatment programs. Persisting symptoms are normalized as experiences that most people may have when they are under extreme biological or psychosocial stress. The personal resources and skills of patients are reinforced, rather than their defects and disabilities. Homework assignments are given to carry out goal-oriented tasks, aided by nursing staff, after the sessions. These are reviewed at the start of the next group the following day. **The training last 1 hour and half and the number of inpatients range from 7 to 14 according to the people admitted.**

### Treatment setting

The group program is conducted by one professional with the assistance of another. They are called the "conductor" and "co-conductor". Conductors come from various professional backgrounds, but in our experience the presence of a doctor (often as a co-conductor) is helpful when discussing detailed information about symptoms, mental state, cognitive impairment, etc. Other professionals on the unit are expected to attend the groups as observers and may be invited to comment by the conductors. White boards are used to make notes during the sessions. Groups are held every weekday from 09.15 to 11.00. The topic for each session is decided at a brief staff meeting prior to the group.

### Structure of sessions

Sessions are clearly structured and follow the steps outlined in the manual [13].

Each session concerns one main theme. These include "*constant themes*" to be provided regardless of the psychopathology of patients, such as: 1) *"What **has occurred **before the admission*", that is used to obtain patients' descriptions about crises and life stresses associated with the crisis leading to admission; 2) "*Stress-vulnerability model*"; 3) "*Drugs*", used for the educational intervention; 4) "*Early warning signs*"; 5) "*Goals after discharge*".

In addition to these topics the following themes are introduced according to the psychopathological status of participants. For this reason they are called "*Optional themes*". They are: 1) "*Alcohol*"; 2) "*Hallucinations*"; 3) "*Anxiety and fear*"; 4) "*Delusions and psychotic thinking*"; 5) "*Mood disorder: sadness and happiness*"; 6) "*Personality disorder cluster A: Anger*"; 7) "*Personality disorder cluster B: secondary advantages*"; 8) "*Suicidal ideas*"; 9) "*Compulsory treatment*".

Every session is structured in the following way: 1) "*Introduction*", to outline the aims and rules of the group session; 2) "*Introduction of all newly admitted patients*"; 3) "*Review of the homework of the previous afternoon*"; 4) "*Summary of the group last session*"; 5) "*The theme of the day*"; 6) "*Summary of the principal points and assigning homework tasks for the afternoon*";

### Rules and group strategies

All rules and strategies are used in a flexible way **and are extensively described in the manual **[13]. Every day before running the group the conductor illustrate to the inpatients the main rules. One of that regard "how and when" they can speak. The other one is that the inpatients are free to leave the session and to come back every where they like to do that. Finally, it is well underlined that if some of them are not able to respect the rules (for istance they frequently interrupt other participants or if they become aggressive) they must leave the group-session and to come back only if they are able to respect these rules.

The main strategies are: 1) socratic questioning, as **described by Perris **[15]; 2) encouraging effective communication among patients and to open dialogue among people with similar conditions rather than professionals giving information and advice to patients [16]; **the first and second strategies are of a crucial importance because promote a high degree of adherence to the approach on the basis of a feeling of "reciprocity"**; 3) helping patients make connections between their thoughts, emotions and behaviors [15]; 4) modelling and role-playing; 5) "normalization" of symptoms and hospital admissions [17]; 6) positive reinforcement and constructive feed-back [18]; 7) structured problem solving **but used in a flexible way **[19,20]; **8) **communication skills [18], **i.e "*positive feelings", "positive remarks", "negative feelings*" and "*active listening*".**

### Evaluation of effectiveness

The results observed during the first and second year of routine application of inpatient CBGT (y1 and y2) are compared with the year before its introduction (y0).

90% of inpatients attended the CBGT sessions. However, in order to compare the specific effectiveness of this approach, patients with the following characteristics were excluded from the evaluation: a) not resident in the catchment area of the mental health department; b) psycho-organic syndromes; c) age > 64 years ; d) length of admission 1 or 2 days; e) bedridden with concomitant physical illness; f) co-morbid substance abuse; g) severe mental retardation; h) admissions from and/or discharges to prison.

Indicators of effectiveness included: 1) frequency of aggressive and violent behaviour in the ward; 2) frequency of readmissions and the proportion admitted on compulsory treatment orders; 3) ward atmosphere; 4) satisfaction of patients and their relatives; 5) satisfaction of professionals.

The evaluation included:

a) **Readmission to hospital, both voluntary and compulsory. **This indicator is crucial because in the absence of community mental health centres in the Molise region the inpatient unit is the only place to manage crises.

b) **Length of hospital stay.**

c) **Patient Satisfaction was **assessed by a self-rated questionnaire, derived from a tool developed by the Italian National Health Institute [21]. The 5 items selected were: 1) "satisfaction with the care received"; 2) "availability of professionals when needed"; 3) "helpfulness of professionals"; 4) "satisfaction with the information received during the admission"; 5) "satisfaction with the treatments received during the admission". For each item there were 5 levels with operational criteria from "few" (level 1) to "very much or always" (level 5). The patients completed the questionnaire before discharge. The evaluation of patient satisfaction began on 1^st ^January 2001. The CBGT started in July 2001. Therefore, this comparison was made between the first 6 months of 2001 *vs *the first 6 months of 2002 and 2003.

d) **Ward atmosphere **was assessed on a scale developed in collaboration with Italian National Health Institute. The inter-rater reliability was carried out on 20 ratings by nurses: agreement exceeded 90% with a Cohen's kappa > .70. The scale rates communication among patients and professionals, the presence/absence of aggressive or violent behavior and bizarre behavior **on a 4-items that are defined with operational and explicit criteria ranging from 1 (that means good atmosphere) to 4 (bad atmosphere). **To facilitate the rating items are also coded by color: 1) *green*, if the atmosphere is acceptable; 2) *yellow*, if there are one or more patients with disturbing behavior that is not alarming; 3) *orange*, if there are one or more patients with disturbing behavior that require immediate interventions but coercion is not necessary; 4) *red*, if there are one or more patients with disturbing behavior that require interventions with coercion and physical restraint.

e) **Use of physical restraints. **Number of times that restraint was used.

### Statistical analysis

Comparisons between parametric variables was performed with analysis of variance and between non-parametric variables with chi square (χ^2^) **and chi square for linear trend where necessary**. The program used was version 11.5 of SPSS for Windows.

## Results

The year before the introduction of CBGT (y0) 280 patients were admitted, during the first year with CBGT (y1) 314, and during the second year (y2) 324. **The diagnosis was made with DSM-IV criteria.**

Schizophrenia and mood disorders were the most common diagnoses of the included and excluded patients (Tables 1 and 2).

**Table 1:** Clinical Characteristics and readmissions of inpatients included in CBGT grouped into categories of diagnosis during the year before CBGT (y0), first and second year (y1, y2) after CBGT.

Patients included in CBGT	"y0"	"y1"	"y2"	P <
Number of patients included in evaluation (% of total admissions)	150 (53%)	171 (54%)	181 (56%)	
Schizophrenic disorder	66 (44%)	62 (36%)	69 (38%)	
Major depressive disorder	20 (13%)	19 (11%)	32 (18%)	
Bipolar disorder	18 (12%)	32 (19%)	22 (12%)	
Personality disorders	18 (12%)	31 (18%)	24 (13%)	
Dual diagnosis	19 (13%)	21 (12%)	24 (13%)	
Anxiety disorders	6 (4%)	3 (2%)	7 (4%)	
Other	3 (2%)	3 (2%)	3 (2%)	
Proportion of patients readmitted	36 (24%)	29 (17%)	25 (15,6%)	.05*
Total number of readmissions	57 (38%)	46 (27%)	43 (24)	.04*
Compulsory readmissions	10 (17%)	2 (4%)	2 (4%)	.03**

* "y2" vs "y0"; ** "y1" and "y2" vs "y0";

**Table 2:** Diagnosis of patient who were excluded from the evaluation during the year before CBGT (y0), the first and second year (y1, y2) after CBGT.

Patients excluded from evaluation	"y0"	"y1"	"y2"
Number excluded from the evaluation (% of all admissions)	130 (47%)	143 (46%)	143 (44%)
Schizophrenic disorders	36 (29%)	34 (24%)	34 (24%)
Major depressive disorders	26 (20%)	29 (20%)	36 (25%)
Bipolar disorders	12 (9%)	11 (8%)	9 (6%)
Personality disorders	5 (4%)	17 (12%)	18 (12%)
Dual Diagnosis	29 (23%)	22 (15%)	27 (19%)
Mental retardation	4 (3%)	5 (3%)	3 (2%)
Anxiety disorders	8 (6%)	12 (8%)	8 (5%)
Dementia	4 (3%)	8 (5%)	8 (5%)
Other	6 (5%)	5 (3%)	3 (2%)

In y0 150 (53% of all admissions) patients were included in the evaluation study; at y1 171 (54%), and at y2 181 (56%). The most frequent causes of exclusion (Table 3), were residence in a different catchment area, aged over 65 years and very brief admissions.

**Table 3:** Frequency of criteria of exclusion from the evaluation of CBGT

Criteria	"y0"	"y1"	"y2"
Admitted but excluded from evaluation	130 (47%)	143 (46%)	143 (44%)
Residence in another catchment area	59 (45%)	66 (46%)	70 (49%)
Age ≥ 65 years	43 (35%)	43 (30%)	49 (34%)
Length of admission of 1–2 days	16 (12%)	15 (11%)	15 (11%)
Diagnosis	8 (6%)	9 (6%)	5 (3%)
Bedridden	2 (1%)	7 (5%)	3 (2%)
Admitted on forensic orders	2 (1%)	3 (2%)	1 (1%)

Thirty-six (24%) had one or more readmissions in y0, 29 (17%) in y1 and 25 (16%) in y2. The reduction y0 vs y2 was significant (χ^2 ^= 3.88, p < .05) **as well as from y2 to y0 (χ^2 ^for linear trend = 5.19; p < .01). **Readmissions (Table I) were 57 (38%) during y0, 46 (27%) during y1, and 43 (24%) during y2. This reduction was significantly lower during year 2 than at baseline (χ^2 ^= 4.19, p < .04).

There were 10 compulsory admissions in y0, 2 in y1, and 1 in y2. The reductions between years 1 and 2 and y0 were significant (χ^2 ^= 4.3, p < .03).

The mean length of stay was reduced from 14 days (y0) to 12.5 days (y1) and 11.5 days (y2). However this change was not significant.

Table 4 shows that satisfaction had increased significantly on all items in y2 compared to y1 and y0.

**Table 4:** Satisfaction of inpatients on the day of discharge. Coded from 1 (a little or not at all) to 5 (too much or always).

Item	"y0" Mean (s.d.)	"y1" Mean (s.d.)	"y2" Mean (s.d.)	p < (Anova)
Care received	4.3 (.7)	4.4 (.8)	4.6 (.8)	F = 6,3 (df = 2)*
Aavailability of professionals when needed by the patient	4.3 (.8)	4.5 (.6)	4.7 (.6)	F = 6,6 (df = 2)*
Helpfulness of professionals	4.3 (.8)	4.4 (.8)	4.7 (.8)	F = 9,9 (df = 2)**
Information received	4.3 (.8)	4.5 (.7)	4.6 (.7)	F = 4,8 (df = 2)*
"Activities" in the ward in the afternoons	3.9 (1)	4.5 (.7)	4.6 (.7)	F = 16,3(df = 2)**

* p < .01 ** p < .001

Ward atmosphere in y0 averaged 2.1 (± 0.8), or code "*yellow*", in y1 the mean was 1.8 (± 0.7), less than yellow, and in y2 it was 1.2 (± 0.7) code "*green*". The difference observed among the three years was statistically significant (F = 73.0; df 2; p < .001).

The use of *physical restraints*, that is rarely used in our unit was carried out 5 times in y0 and once in each of the following two years. The code "red" was never used throughout y1 and y2.

## Discussion

**For what concern the methodology this is not a randomized controlled trial but it is based on "historical controls" that limits, of course, to generalize the results. The choice has been that to do an effective study with the relatively long follow-up and completeness of the data add weight to the findings. **The variables evaluated appear pertinent to the aim of the study and suggest an improvement in the quality of treatment of acute psychiatric crises. In fact, this study of the effectiveness of psychosocial treatment in the form of daily cognitive behavioural group therapy on the outcome indicators of an acute inpatient unit suggests that wide ranging benefits may be achieved. These include reduced readmission rates, improved ward atmosphere and patient satisfaction. Compulsory admissions and violent episodes were minimized.

We have considered indicators of *results *(average length of stay), of *process*, according to Donabedian's Theory [22], of *quality evaluation *(ward atmosphere, physical restraints), of *process-outcome *(readmissions, including compulsory admissions), and of subjective *outcome *(patient satisfaction).

Direct clinical indicators, such as reductions in clinical and social morbidity, were not measured. Lack of external funding to provide independent ratings prevented us from conducting formal assessments of this kind. This structured group approach took the place of the traditional personal relationship between patients and doctors, often without involving nurses and other professionals. As a consequence we were able to improve the efficiency. In 1.5 hours all patients could be observed by the medical staff in the presence of nurses and other staff. This appeared to result in better collaboration among all professionals. Personal clinical interviews with patients were not forbidden; but in our opinion the CBGT facilitates interpersonal work and helps clarify and achieve the goals of inpatient treatment. In this way it also improved training in self-management of mental disorders that may be learned more successfully in the acute phase than in the community when the clinical condition of patients has stabilized and they may have less motivation to learn these strategies. In the years before the introduction of CBGT, the interpersonal clinical approach and optimal pharmacotherapy did not reduce the "*revolving door*" or the length of stay. After the introduction of this approach we were able to reduce the number of inpatient beds from 15 to 12 (1.15 per 10,000 to 0.9 per 10,000) and we reallocated those three beds to the day-hospital.

It could be argued that the improvements are the consequence of factors not considered in this study. For example better outpatient treatment or advances in pharmacotherapy. However, in the three years of the study nothing changed in the organization of the services and no major innovations in drugs or other psychosocial treatments were introduced.

Bazzoni and colleagues [10], using a similar approach, **but without a manual **[13]**so structured**, achieved similar significant reductions in readmissions (12.2%from 16.9%), proportions of compulsory readmissions (25% from 72%), and violent episodes (24.7% from 41.5%) in the first year of CBGT compared to the previous year. The combined results of these two Italian projects that employed very similar approaches lend weight to the conclusion that CBGT may be a valuable intervention for acute inpatients.

To date, randomized controlled studies of cognitive behavioural therapies in acute inpatient settings have shown equivocal benefits. Drury and colleagues [23,24] reported an individual and group approach that reduced the time to remission of psychotic symptoms. However, an attempted replication of this study using improved methodology did not achieve any significant benefits over a two-year follow-up [25]. A more recent study of individual cognitive behavioural therapy for inpatients with recent onset schizophrenic disorders showed improved rates of remission, but little specific benefits compared to supportive counselling or pharmacotherapy at 6-week or 18-month follow-up [26]. Recurrences and readmissions were similar in all groups. All these studies focused on psychotic symptoms and lacked the broader biopsychosocial perspective employed in our cognitive behavioural group sessions. **We are aware that the promising results we have obtained in a consistent manner over several years must be replicated in well-controlled trials to consider their clinical efficacy. At the same time our general conclusion is that this intervention is effective (i.e., less re-admissions and above all compulsory admissions), efficient (reduction of the mean length of stay and of the number of beds) with a high degree of the satisfaction of admitted. Finally, in our opinion, the key effective factors are: 1) a dimensional as well as a stress/vulnerability model of illness; 2) a sharing process of cure based on the strategies of socratic questioning, of avoidance of "up-down" communication (that both encourage a feeling of reciprocity) and of normalization of symptoms as well as of hospitalization; 3) psychoeducational techniques; 4) problem-solving approach even if it is used in a flexible way.**
